# Supplementary material for: Two-stage electro–mechanical coupling of a KV channel in voltage-dependent activation
Source: Nat Commun. 2020 Feb 3;11:676. doi: 10.1038/s41467-020-14406-w (PMC6997178; doi:10.1038/s41467-020-14406-w)
Supplement: Supplementary file 1 — Supplementary Information [file 41467_2020_14406_MOESM1_ESM.pdf]

***Supplementary files for:***

**Two-stage electro-mechanical coupling of a $K_V$ channel in voltage-dependent activation**

Panpan Hou, Po Wei Kang, Audrey Deyawe Kongmeneck, Nien-Du Yang,

Yongfeng Liu, Jingyi Shi, Xianjin Xu, Kelli McFarland White, Mark A. Zaydman, Marina A. Kasimova, Guiscard Seebohm, Ling Zhong, Xiaoqin Zou, Mounir Tarek*, and Jianmin Cui*

*To whom correspondence should be addressed. E-mail: jcui@wustl.edu (J.C.) or mounir.tarek@univ-lorraine.fr (M.T.)

**This PDF File Includes:**

Supplementary Table 1

Supplementary Figures. 1 to 7

**Supplementary Table 1. All primer sequences used in this study.**

| Mutations | Primer sequences |
|---|---|
| **C214A** | pB: cttggaTcccacCGCgaggaccacca<br>pC: cGCGgtgggAtccaaggggcaggtg |
| **G219C** | pB: cctgaCacttggaTcccacCGC<br>pC: ggAtccaagtGtcaggtgtttgccacg |
| **C331A** | pB: gacagagaaTGCggaggcgatggtcttc<br>pC: ctccGCAttctctgtctttgccatc |
| **E160R** | pB: cgatTCTcatccagaagagagt<br>pC: ctcttctggatgAGAatcgtgctggtggtgttct |
| **R231E** | pB: caggaaTTCgatgcccctgatggc<br>pC: gggcatcGAAttcctgcagatcctgag |
| **R237E** | pB: gcatcTCcaggatctgcaggaag<br>pC: cagatcctgGAgatgctacacgtcgac |
| **F127A** | pB: gaagacggcgGCAtggtaaacgaagcatttc<br>pC: caTGCcgccgtcttcctcatc |
| **K183N** | pB: cacgtaAttgctgcggcagcc<br>pC: ccgcagcaaTtacgtgggcctctgg |
| **R190Q** | pB: gcagcTgcccccagaggcc<br>pC: ctgggggcAgctgcgctttgccc |
| **F193A** | pB: cgTgcaGCgcgcagccgcccc<br>pC: ctgcgcGCtgcAcggaagcccatttcc |
| **R195A** | pB: ggcttAGCggcaaagcgcagccg<br>pC: gctttgccGCTaagcccatttccatcatc |
| **K196E** | pB: ctgcgctttgcccggGagcccatttc<br>pC: ccggGagcccatttccatcatc |
| **P197A** | pB: ggaaatTGCcttccgggcaaagcg<br>pC: ccggaagGCAatttccatcatcgacctc |
| **R237W** | pB: gcatccAcagTatctgcaggaagcgg<br>pC: cagatActgTggatgctacacgtcg |
| **Q244W** | pB: cctccccaccggtcgacgtgtagc<br>pC: gaccggtggggaggcacctggag |
| **G245W** | pB:ccaggtcccccactggcggtcgacgtg<br>pC:cagtgggggacctggaggctcct |
| **G246W** | pB: ccaggtccaaccctggcggtcgac<br>pC:cagggttggacctggaggctcctg |
| **T247W** | pB: gagtctccaccagcctccctggcggtc<br>pC: ggctggtggagactcctgggctccgtg |
| **W248R** | pB: cctccGggtgcctccctggc<br>pC: gaggcaccCggaggctcctgggc |
| **R249W** | pB: gagccAccaCgtgcctccctggcg<br>pC: ggcacGtggTggctcctgggctcc |
| **L250W** | pB: gagccGagCCAcctccaggtgcctc<br>pC: gaggTGGctCggctccgtggtcttc |

| | |
|---|---|
| **L251A** | pB: cggaTcccGCgagcctccaggtgcc<br>pC: gctcGCgggAtccgtggtcttcatcc |
| **G252W** | pB: gaccacTgaCcAcaggagcctccaggtg<br>pC: cctgTgGtcAgtggtcttcatccaccg |
| **S253W** | pB: gaccacCCagcccaggagcctcc<br>pC: gggctGGgtggtcttcatccacc |
| **V254M** | pB: gaagaccaTggagcccaggagcc<br>pC: ggctccAtggtcttcatccaccg |
| **V255W** | pB: gatgaaCCAcacggagcccaggag<br>pC: ctccgtgTGGttcatccaccgccagg |
| **F256A** | pB: gtggatgGCgaccacggagcccag<br>pC: gtggtcGCcatccaccgccagg |
| **I257W** | pB: gcggtgCCAAaagaccacggagccc<br>pC: ggtcttTTGGcaccgccaggagctg |
| **H258W** | pB: ctggcgCCAgatgaagaccacggag<br>pC: cttcatcTGGcgccaggagctgataac |
| **R259W** | pB: cagctcctgCcAgtggatgaagaccacgg<br>pC: cacTgGcaggagctgataaccac |
| **Q260W** | pB: gctccCACcggtggatgaagaccac<br>pC: ccaccgGTGggagctgataaccacc |
| **E261K** | pB: gttatAagctTctggcggtggatgaag<br>pC: cgccagAagctTataaccaccctgtacatc |
| **L262A** | pB: ggtggtAatTGCctcctggcggtggatg<br>pC: ggagGCAatTaccaccctgtacatcg |
| **I263A** | pB: ggtggttGCcagctcctggcggtg<br>pC: gagctgGCaaccaccctgtacatc |
| **T264A** | pB: cagTgtggCtatcagctcctggcg<br>pC: ctgataGccacActgtacatcg |
| **T265A** | pB: gtacagAgCggttatcagctcctg<br>pC: gataaccGcTctgtacatcggcttc |
| **L266A** | pB: GATAACCACCGCCTACATCG<br>pC: CGATGTAGGCGGTGGTTATC |
| **Y267A** | pB: ccgatAGCcagggtggttatcagc<br>pC: caccctgGCTatcggcttcctgggcc |
| **I268W** | pB: gaagccCCAgtacagggtggttatcag<br>pC: cctgtacTGGggcttcctgggcctc |
| **G269A** | pB: CTGTACATCGCCTTCCTGGGCCT<br>pC: AGGCCCAGGAAGGCGATGTACAG |
| **F270A** | pB: GTACATCGGCGCCCTGGGCCTC<br>pC: GAGGCCCAGGGCGCCGATGTAC |
| **L271E** | pB: gatgaggccTTCgaagccgatgtacagg<br>pC: cttcGAAggcctcatcttctcc |
| **G272A** | pB: CTTCCTGGCCCTCATCTTCT<br>pC: AGAAGATGAGGGCCAGGAAG |
| **L273F** | pB: gaagatgaATcccaggaagccgatg<br>pC: cctgggATtcatcttctcctcgtac |

| | |
|---|---|
| **I274A** | pB: TGGGCCTCGCCTTCTCGAGCTA<br>pC: TAGCTCGAGAAGGCGAGGCCCA |
| **F275A** | pB: GCCTCATCGCCTCGAGCTA<br>pC: TAGCTCGAGGCGATGAGGC |
| **F335A** | pB: CTTCTCTGTCGCTGCCATCTCC<br>pC: CTTCTCTGTCGCTGCCATCTCC |
| **A336G** | pB: gaaggaAatTCcaaagacagagaagcag<br>pC: gtctttgGAatTtccttctttgcgctcc |
| **I337A** | pB: CTGTCTTTGCCGCCTCCTTCTTTGC<br>pC: GCAAAGAAGGAGGCGGCAAAGACAG |
| **S338F** | pB: gaagAATatTgcaaagacagagaag<br>pC: gtctttgcAatATTcttctttgcgctccc |
| **F339S** | pB: gcaaaACTggagatggcaaagacag<br>pC: ccatctccAGTtttgcgctcccagcg |
| **F340A** | pB: GCCATCTCCTTCGCTGCGCTCCCA<br>pC: TGGGAGCGCAGCGAAGGAGATGGC |
| **A341V** | pB: gggagcAcaaagaaggagatggc<br>pC: ccttctttgTgctcccagcgggg |
| **L342A** | pB: CTTCTTTGCGGCCCCAGCG<br>pC: CGCTGGGGCCGCAAAGAAG |
| **P343A** | pB: ccgcGgCgagcgcaaagaaggag<br>pC:gcgctcGcCgcggggattcttggc |
| **A344V** | pB: gaatccccActgggagcgcaaagaag<br>pC: ctcccagTggggattcttggctcg |
| **G345A** | pB: gccaagaatcGccgctgggagcgc<br>pC: gcggCgattcttggctcggg |
| **I346A** | pB: gagccaagGGCccccgctgggagcgc<br>pC: gggGCCcttggctcggggtttg |
| **L347A** | pB: gagccaGCaatccccgctgggagc<br>pC: ggggattGCtggctcggggtttgcc |
| **G348A** | pB: ccgaCGcaagaatccccgctg<br>pC: ggattcttgCGtcggggtttgccctg |
| **S349A** | pB: gcaaaccccgCgccaagaatccccgc<br>pC: ggcGcggggtttgccctg |
| **G350A** | pB: ggcaaacGccgagccaagaatcc<br>pC: gctcggCgtttgccctgaaggtg |
| **F351A** | pB: ggcaGCGcccgagccaagaatcc<br>pC: gctcgggCGCtgccctgaaggtgcag |
| **A352C** | pB: GATCCGGGTTTTGCCTGAAGGTGCAG<br>pC: CTGCACCTTCAGGCAAAACCCGGATC |
| **L353K** | pB: ccttcTTggcaaaccccgagc<br>pC: gggtttgccAAgaaggtgcagcagaag |
| **K354A** | pB: ctgcacGGCcagggcaaaccccg<br>pC: ccctgGCCgtgcagcagaagcag |
| **V355W** | pB: gggtttgccctgaagTGgcagc<br>pC: gggtttgccctgaagTGgcagc |

| | |
|---|---|
| **Q356A** | pB: GTTTGCCCTGAAGGTGGCGCAGAAGCAG<br>pC: CTGCTTCTGCGCCACCTTCAGGGCAAAC |
| **Q357A** | pB: GAAGGTGCAGGCGAAGCAGAGGCAGA<br>pC: TCTGCCTCTGCTTCGCCTGCACCTTC |
| **K358A** | pB: GTGCAGCAGGCGCAGAGGCAGAAGCAC<br>pC: GTGCTTCTGCCTCTGCGCCTGCTGCAC |
| **Q359A** | pB: GTGCAGCAGAAGGCGAGGCAGAAGCAC<br>pC: GTGCTTCTGCCTCGCCTTCTGCTGCAC |
| **R360A** | pB: CAGCAGAAGCAGGCGCAGAAGCACTTC<br>pC: GAAGTGCTTCTGCGCCTGCTTCTGCTG |
| **M238W** | pB: gacgtgCagcCAcctcaggatctgcagg<br>pC: gaggTGgctGcacgtcgaccgccag |
| **E160R/ R237E/ M238W** | Use E160R/R237E as template<br>M238W-pB: gacgtgCagcCAcTCcaggatctgcagg<br>M238W-pC: GAgTGgctGcacgtcgaccgccag |
| **L239W** | pB: cgacgtgCCAcatcctcaggatctgc<br>pC: ggatgTGGcacgtcgaccgccag |
| **E160R/ R237E/ L239W** | Use E160R/R237E as template<br>L239W-pB: gacgtgCCAcatcTCcaggatctgc<br>L239W-pC: GAgatgTGGcacgtcgaccgccag |
| **D242W** | pB: gcgCCAgacgtgtagcatcctc<br>pC: ctacacgtcTGGcgccagggaggcac |
| **R243W** | pB: ccctgCcAgtcgacgtgtagcatc<br>pC: gtcgacTgGcagggaggcacctgg |
| **I268A** | pB: caggaaTccgGCgtacagggtggttatcag<br>pC: gtacGCcggAttcctgggcctcatcttc |
| **L251F** | pB: gagccGaAgagcctccaggtgcc<br>pC: gaggctcTtCggctccgtggtcttc |
| **F339L** | pB: cgcGaaGaGggagatggcaaagacag<br>pC: catctccCtCttCgcgctcccagcggg |

## Supplementary Figures

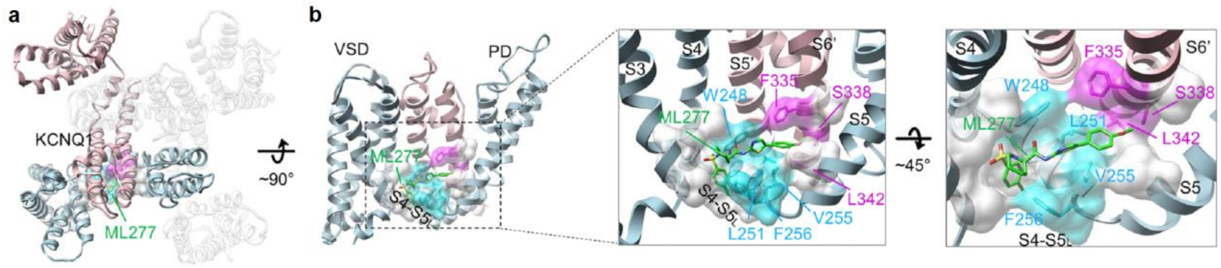


**Supplementary Fig. 1, Molecular docking results of ML277 onto KCNQ1 channel.**

**(a)** Residues interacting with ML277 based on molecular docking and experimental results. Top view of the binding mode of ML277 (green) at the homology model of $hK_V7.1$ (see Methods). Two neighboring subunits (light red and light blue) are highlighted. **(b)** Side view of the binding mode of ML277. Only two neighboring subunits (light red and light blue) are shown for clarity. Right panels, detailed structure of the ML277 (green) binding at the S4-S5L/pore interface. Interacting residues that consistent with mutagenesis results shown in cyan (S4-S5L) and magenta (pore).

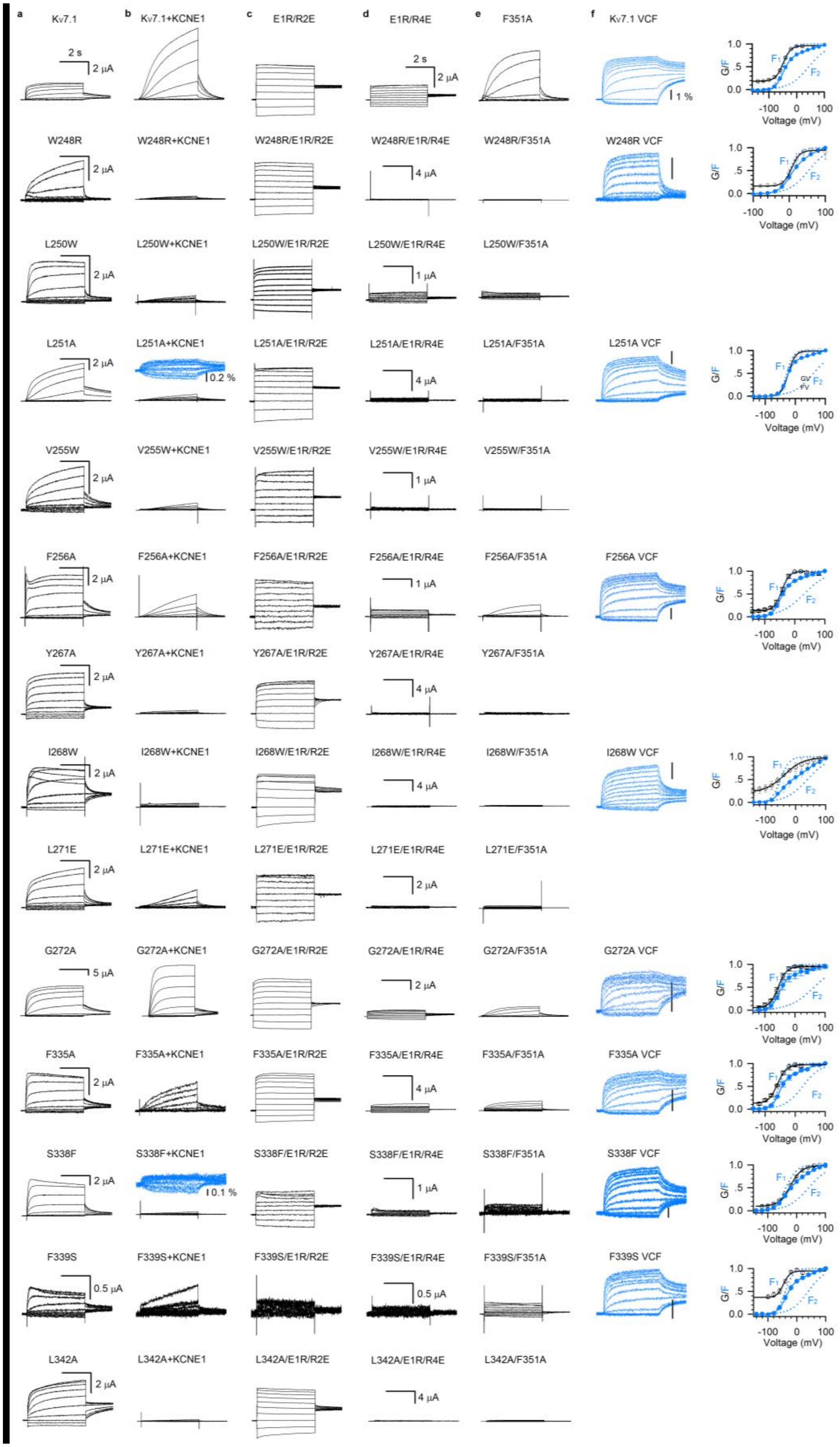
a Kv7.1
b Kv7.1+KCNE1
c E1R/R2E
d E1R/R4E
e F351A
f Kv7.1 VCF
W248R
W248R+KCNE1
W248R/E1R/R2E
W248R/E1R/R4E
W248R/F351A
W248R VCF
L250W
L250W+KCNE1
L250W/E1R/R2E
L250W/E1R/R4E
L250W/F351A
L251A
L251A+KCNE1
L251A/E1R/R2E
L251A/E1R/R4E
L251A/F351A
L251A VCF
V255W
V255W+KCNE1
V255W/E1R/R2E
V255W/E1R/R4E
V255W/F351A
F256A
F256A+KCNE1
F256A/E1R/R2E
F256A/E1R/R4E
F256A/F351A
F256A VCF
Y267A
Y267A+KCNE1
Y267A/E1R/R2E
Y267A/E1R/R4E
Y267A/F351A
I268W
I268W+KCNE1
I268W/E1R/R2E
I268W/E1R/R4E
I268W/F351A
I268W VCF
L271E
L271E+KCNE1
L271E/E1R/R2E
L271E/E1R/R4E
L271E/F351A
G272A
G272A+KCNE1
G272A/E1R/R2E
G272A/E1R/R4E
G272A/F351A
G272A VCF
F335A
F335A+KCNE1
F335A/E1R/R2E
F335A/E1R/R4E
F335A/F351A
F335A VCF
S338F
S338F+KCNE1
S338F/E1R/R2E
S338F/E1R/R4E
S338F/F351A
S338F VCF
F339S
F339S+KCNE1
F339S/E1R/R2E
F339S/E1R/R4E
F339S/F351A
F339S VCF
L342A
L342A+KCNE1
L342A/E1R/R2E
L342A/E1R/R4E
L342A/F351A
2 s
2 μA
1 μA
4 μA
5 μA
0.5 μA
1 %
0.2 %
0.1 %
G/F
Voltage (mV)
F1
F2
GV
FV

**Supplementary Fig. 2. Dataset for functional validations of AO state E-M coupling residues identified by the ML277 screen.**

**(a-b)** Currents of WT and mutant $K_V$7.1 with and without KCNE1. Currents are shown in the same scale. VCF experiments was performed on S338F+KCNE1 and L251A+KCNE1, indicating membrane expression and VSD function. **(c-e)** Currents of WT and mutant $K_V$7.1 on top of the IO background E1R/R2E, the AO background E1R/R4E and F351A. Currents are shown in the same scale. **(f)** VCF results of WT and eight mutations in $K_V$7.1*. $n \geq 3$. Source data are provided as a Source Data file.

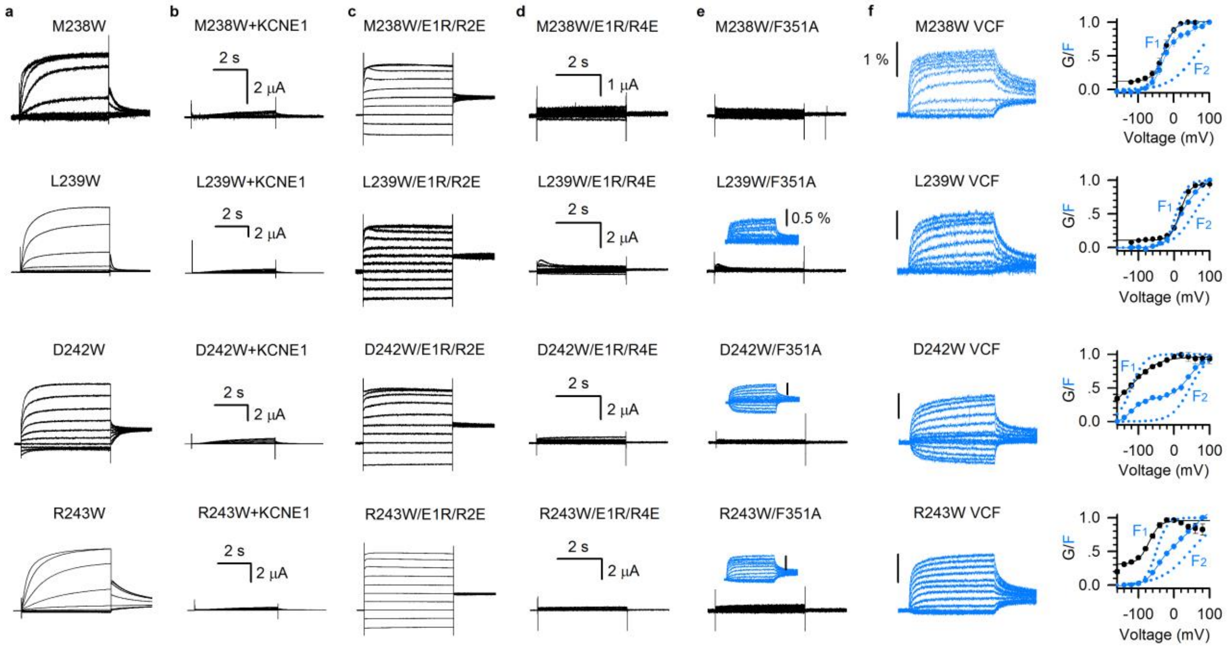


**Supplementary Fig. 3. Dataset for functional validations of S4c residues critical for AO state E-M coupling.**

**(a-b)** Currents of S4c mutations with and without KCNE1. Currents are shown in the same scale. **(c-e)** Currents of the S4c mutations on top of the IO background E1R/R2E, the AO background E1R/R4E and F351A. Currents are shown in the same scale. VCF results (three out of four) are shown in blue. **(f)** VCF results of the four mutations in S4c in $K_V7.1^*$. $n \geq 3$. Source data are provided as a Source Data file.

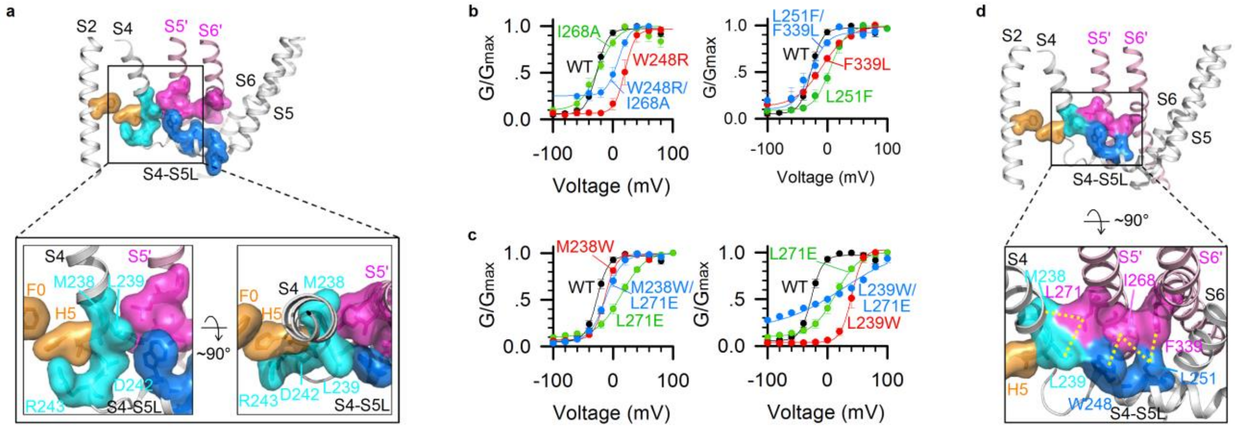


**Supplementary Fig. 4. Double mutant cycle analysis.**

**(a)** Mapping the key residues on to the Kv7.1 cryoEM structure, the VSD of which is at the activated state. Cyan, S4c residues (M238, L239, D242, and R243); Orange, F0 and H5 in the charge transfer center; Blue, S4-S5L residues; Pink, pore residues. Lower panels, detailed structure of the key residues. **(b-c)** Double mutant cycle analysis of the interactions at S4-S5L/pore interface: W248 and I268 (ΔΔG= 1.4 kcal/mol) and L251 and F339 (ΔΔG= 2.7 kcal/mol), as well as the S4c/S5 interface: M238 and L271 (ΔΔG= 1.7 kcal/mol) and L239 and L271 (ΔΔG= 4.4 kcal/mol). G–V relations from WT and mutations are fitted with Boltzmann equation. n ≥ 3. **(d)** Mapping the interactions M238/L271 and L239/L271 at the S4c/S5 interface, and W248/I268, L251/I268, and L251/F339 at the S4-S5L/pore interface. Color code is the same as in a, and yellow dashed lines indicate the interactions.

**a**

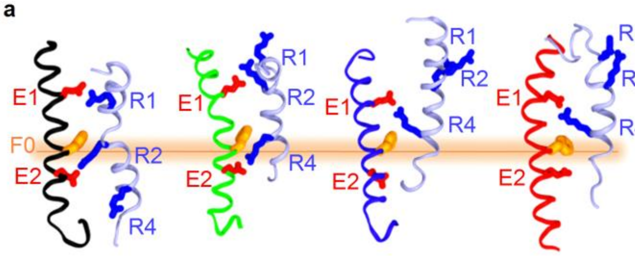


**b**

| State-dependent salt bridges in Kv7.1 models | | | | |
|---|---|---|---|---|
| Salt bridge | Number of subunits (over 4) | | | |
| | RC | IO | AO | AC |
| E1/R1 | 3 | 0 | 0 | 0 |
| E2/R2 | 3 | 0 | 0 | 0 |
| E1/R2 | 1 | 4 | 0 | 0 |
| E2/R4 | 0 | 3 | 0 | 0 |
| E1/R4 | 0 | 0 | 4 | 4 |

**c**

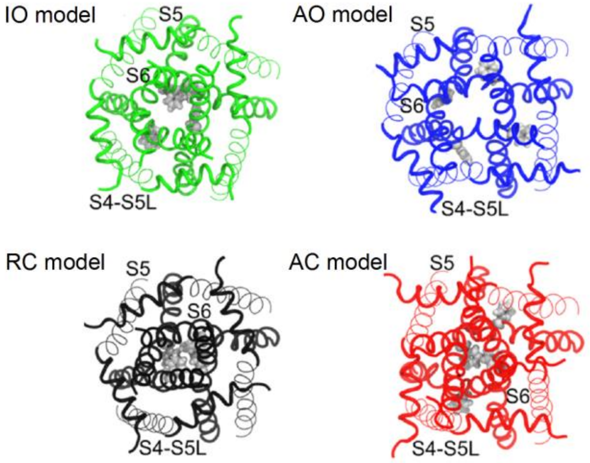


**d**

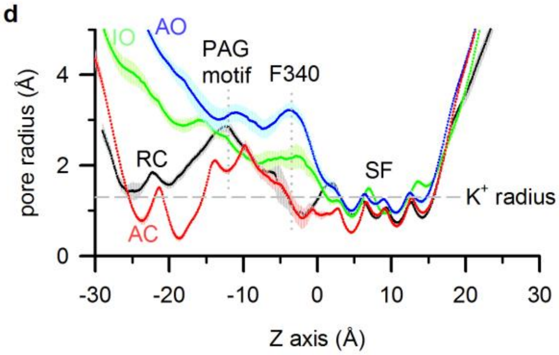


**e**

| Neighbor residues in Kv7.1 models | | | | |
|---|---|---|---|---|
| Residue pairs | Number of subunits (over 4) | | | |
| | RC | IO | AO | AC |
| T144/S298 | 4 | 3 | 4 | 4 |
| T144/Y299 | 4 | 4 | 4 | 4 |
| T144/A300 | 4 | 3 | 4 | 4 |
| F232/F279 | 3 | 4 | 4 | 4 |
| L250/L353 | 4 | 4 | 4 | 0 |
| V254/L353 | 4 | 4 | 4 | 4 |
| H258/L353 | 4 | 4 | 4 | 4 |

**f**

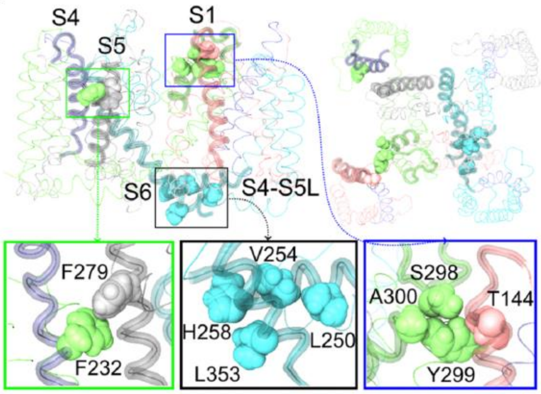


**g**

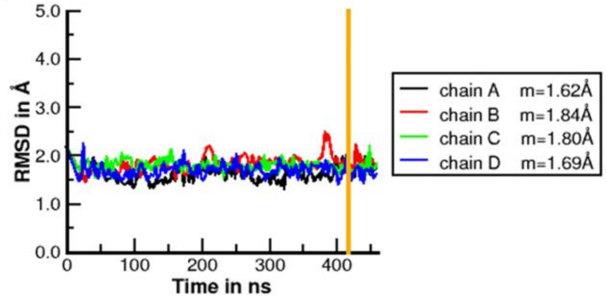


**h**

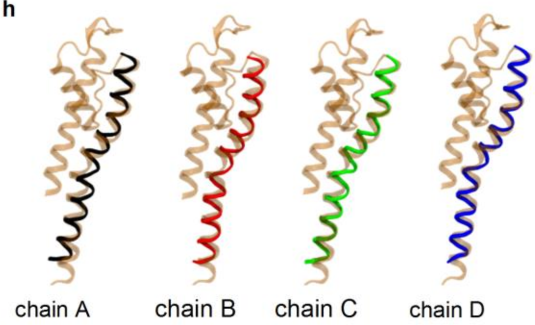

**Supplementary Fig. 5. Structural validation of the MD trajectories obtained for $K_V$7.1 models.**

**(a)** State-dependent $K_V$7.1 VSD salt-bridges[1-4]. Representation of S2 (in black, green, blue and red) and S4 (in blue) segments in $K_V$7.1 RC, IO, AO and AC models, respectively. S4 gating charges are represented in blue sticks, while its S2 countercharges are represented in red sticks. The hydrophobic plug F167 (F0) is represented in orange sticks. **(b)** Summary table of state-dependent $K_V$7.1 VSD salt-bridges in the subunits of $K_V$7.1 models. **(c)** Intracellular view of S4-S5L and PD segments in $K_V$7.1 IO (top, left), AO (top, right), RC (bottom, left) and AC (bottom, right) models. The gray spheres are sidechains of F340. **(d)** Average pore radii of the conduction pathways of $K_V$7.1 models. Standard deviations are represented by black, green, blue and red horizontal bars for the MD trajectories obtained from RC, IO, AO and AC models, respectively. **(e)** Summary table of state-dependent KCNQ1 neighbor residues pairs identified by cysteine scanning studies and previous double mutant cycle analysis in the subunits of KCNQ1 RC, IO, AO and AC models involving T144 [5], F232 [6], and L353 [7]. In this analysis we calculated the distance between Cβ atoms of the residue pairs with a cut-off of 13 Å to determine if these residues are prone to interact when mutated into Cys. **(f)** Interacting pairs of KCNQ1 neighbor residues. In the upper panel, the side view (left) and the extracellular view (right) of the seven pairs of neighbor residues in the KCNQ1 IO model are shown. The transmembrane segments (in cartoon) bearing the residues of interest are highlighted in transparent colors. In the lower panel, the zoomed views of IO state specific interaction in IO model (left), RC state specific interactions in RC model (middle), and of AO state specific residue pairs in AO model (right) are displayed. **(g-h)** Comparison of the pore domain of $K_V$7.1 RC model with $KCNQ1_{EM}$ structure. **(g) T**he graph project the root-mean-square deviation (RMSD) of the pore domain segments of $K_V$7.1 RC model with respect to $KCNQ1_{EM}$ structure throughout its MD simulation time (in ns). Average RMSD values are displayed for each subunit (labeled chain A, B, C, D). The orange vertical line represents the simulation time at which the pictures displayed in (h). **(h)** A cartoon representation of the structural alignment of the S6 segment of $KCNQ1_{EM}$ structure (in transparent orange) with those of $K_V$7.1 (top) RC subunits, each captured at ~420 ns. Each subunit is represented in solid black, red, green and blue cartoon, respectively. Source data are provided in Source Data file.

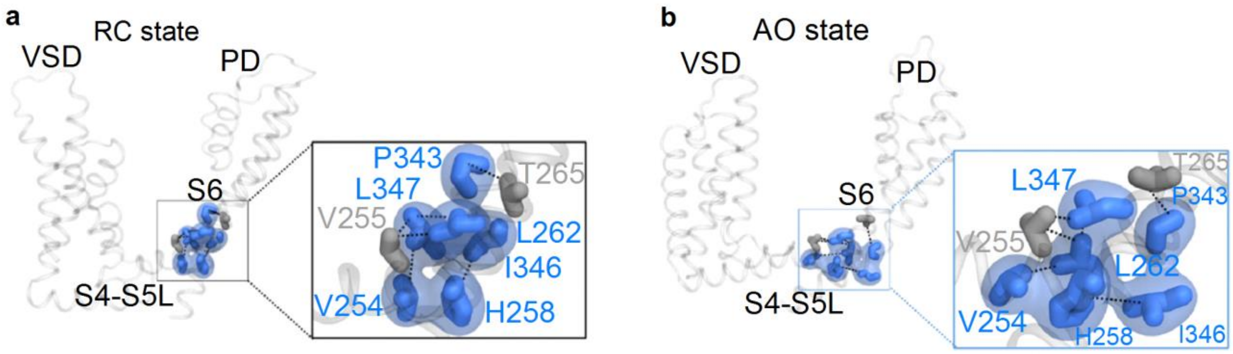


**Supplementary Fig. 6. Possible intra-subunit interactions at the classical E-M coupling region in RC and AO states.**

**(a-b)** Representations of the IO and AO state models of a $K_V7.1$ subunit. Inset: enlarged structures of the boxed area. Blue, residues identified as important for E-M coupling in experiments (Fig. 1c-g); grey: V255 and T265, which were identified in MD simulations as important for E-M coupling, but for which mutations still show functional currents (Fig. 3c). Residue V255 was found to be important for the AO state E-M coupling (Fig. 3). Each pair of residues is represented by a black dashed line connecting their respective sidechains.

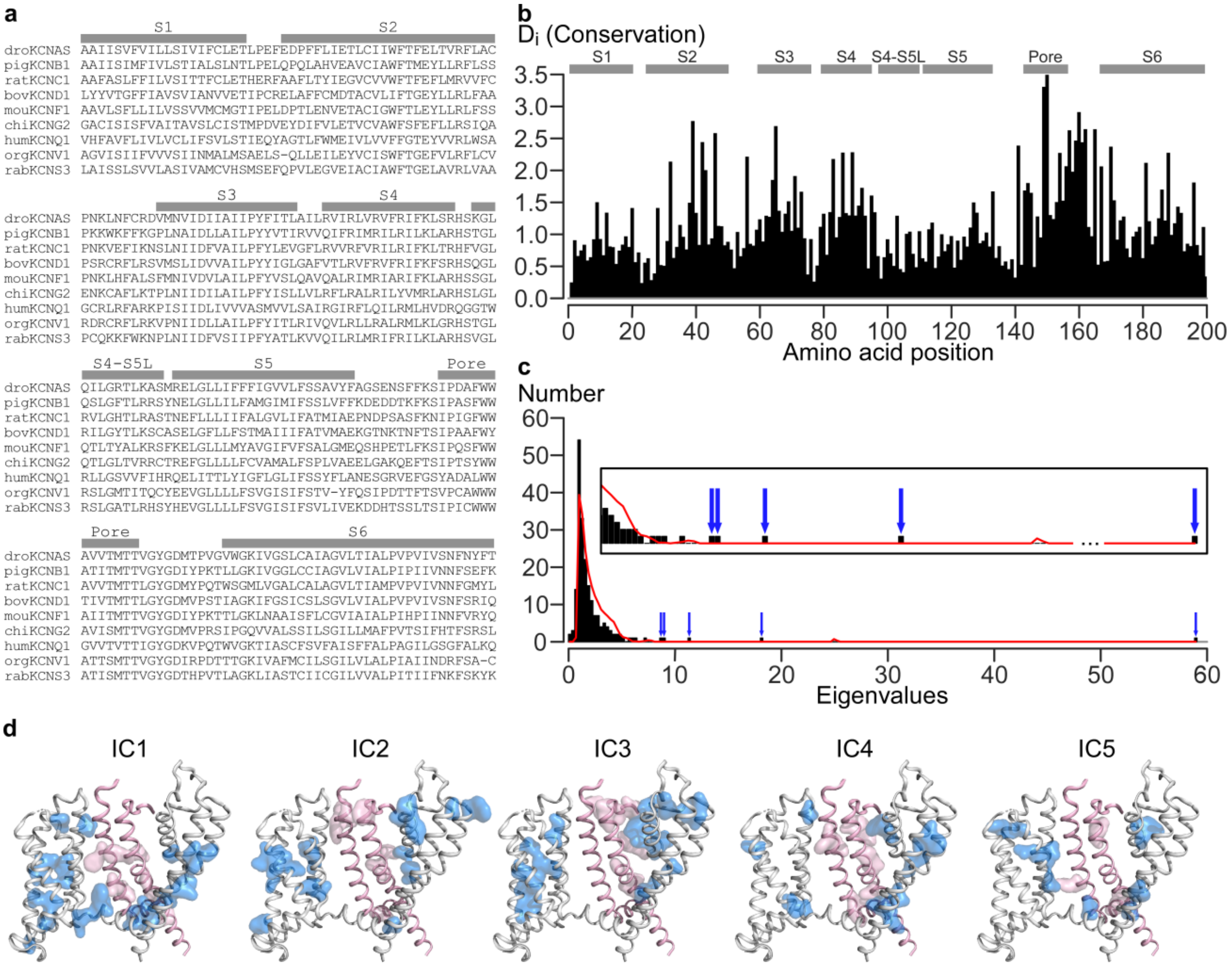


**Supplementary Fig. 7. Statistical coupling analysis of domain-swapped $K_V$ channels.**

**(a)** Expanded sample MSA (from Figure 6a) used as input for analysis by SCA. MSA uses the nomenclature established by the HUGO Gene Nomenclature Committee (HGNC) for voltage-gated potassium channels[8]. $K_V$ channels can adopt two types of architectures. In "domain-swapped" channels, the VSD from one subunit contacts the PD from the neighboring subunit. In "non-domain-swapped" channels, the VSD from one subunit contacts the PD from the same subunit. These distinct architectures likely give rise to different modes of E-M coupling. SCA was applied to the $K_V$ channel superfamily previously[9]; however, prior studies utilized both domain-swapped and non-domain swapped $K_V$ channels in the input data. In this MSA, only domain-swapped $K_V$ channels were included. One member from each of $K_V1$ – $K_V9$ is shown. The final processed MSA includes 1421 sequences at 200 positions. **(b)** First order conservation by amino acid position within the MSA. $D_i$ on the y-axis is calculated based on Kullback-Leibler divergence which measures deviation of the observed amino acid frequency in the MSA (Fig. 6a)

from the natural "background" amino acid frequency (see Methods). Bars indicate positions of the helical segments. **(c)** A histogram of eigenmodes tallied by their associated eigenvalues from SCA calculation (black). Red indicates the spectrum calculated by randomizing the input. Five significant eigenmodes can be seen by the black bars rising above the randomized red curve (blue arrows) at high eigenvalues. Inset shows enlarged scales. **(d)** Amino acid residues within independent component 1 through independent component 5 identified by SCA mapped onto the $K_V$7.1 cryoEM structure. Blue surfaces indicate all IC positions on one subunit, pink surfaces indicate the same IC positions on the neighboring subunit. Only the S5 and S6 helices are shown on the neighboring subunit for sake of clarity (pink helices). Accordingly, only the IC positions on the neighboring subunit on S5 and S6 are shown (pink surfaces). Using the IC submatrix and based on the strength of external correlation (see Methods, Figure 6c), IC2+IC3 are combined to form sector 1 (Fig. 6d), while IC1+IC4+IC5 are combined to form sector 2 (Fig. 6e). Source data for SCA are provided in Source Data file.

## References


1. Hou, P. et al. Inactivation of KCNQ1 potassium channels reveals dynamic coupling between voltage sensing and pore opening. *Nat Commun* **8**, 1730 (2017).
2. Hou, P., Shi, J., White, K. M., Gao, Y. & Cui, J. ML277 specifically enhances the fully activated open state of KCNQ1 by modulating VSD-pore coupling. *Elife* **8** (2019).
3. Zaydman, M. A. et al. Domain-domain interactions determine the gating, permeation, pharmacology, and subunit modulation of the IKs ion channel. *Elife* **3**, e03606 (2014).
4. Wu, D., Pan, H., Delaloye, K. & Cui, J. KCNE1 remodels the voltage sensor of Kv7.1 to modulate channel function. *Biophys J* **99**, 3599-608 (2010).
5. Xu, Y. et al. Building KCNQ1/KCNE1 channel models and probing their interactions by molecular-dynamics simulations. *Biophys J* **105**, 2461-73 (2013).
6. Nakajo, K. & Kubo, Y. Steric hindrance between S4 and S5 of the KCNQ1/KCNE1 channel hampers pore opening. *Nat Commun* **5**, 4100 (2014).
7. Choveau, F. S. et al. KCNQ1 channels voltage dependence through a voltage-dependent binding of the S4-S5 linker to the pore domain. *J Biol Chem* **286**, 707-16 (2011).
8. Gutman, G. A. et al. International Union of Pharmacology. LIII. Nomenclature and molecular relationships of voltage-gated potassium channels. *Pharmacol Rev* **57**, 473-508 (2005).
9. Lee, S. Y., Banerjee, A. & MacKinnon, R. Two separate interfaces between the voltage sensor and pore are required for the function of voltage-dependent K(+) channels. *PLoS Biol* **7**, e47 (2009).
