# Supplementary material for: Two-stage electro–mechanical coupling of a KV channel in voltage-dependent activation
Source: Nat Commun. 2020 Feb 3;11:676. doi: 10.1038/s41467-020-14406-w (PMC6997178; doi:10.1038/s41467-020-14406-w)
Supplement: Supplementary file 3 — Reporting Summary [file 41467_2020_14406_MOESM3_ESM.pdf]

nature research

Corresponding author(s): Jianmin Cui, Mounir Tarek
Last updated by author(s): Dec 10, 2019

Reporting Summary

Nature Research wishes to improve the reproducibility of the work that we publish. This form provides structure for consistency and transparency in reporting. For further information on Nature Research policies, see Authors & Referees and the Editorial Policy Checklist .

Statistics

For all statistical analyses, confirm that the following items are present in the figure legend, table legend, main text, or Methods section.

n/a	Confirmed	
☐	☒	The exact sample size (*n*) for each experimental group/condition, given as a discrete number and unit of measurement
☐	☒	A statement on whether measurements were taken from distinct samples or whether the same sample was measured repeatedly
☐	☒	The statistical test(s) used AND whether they are one- or two-sided<br>*Only common tests should be described solely by name; describe more complex techniques in the Methods section.*
☒	☐	A description of all covariates tested
☐	☒	A description of any assumptions or corrections, such as tests of normality and adjustment for multiple comparisons
☐	☒	A full description of the statistical parameters including central tendency (e.g. means) or other basic estimates (e.g. regression coefficient) AND variation (e.g. standard deviation) or associated estimates of uncertainty (e.g. confidence intervals)
☒	☐	For null hypothesis testing, the test statistic (e.g. *F*, *t*, *r*) with confidence intervals, effect sizes, degrees of freedom and *P* value noted<br>*Give P values as exact values whenever suitable.*
☒	☐	For Bayesian analysis, information on the choice of priors and Markov chain Monte Carlo settings
☒	☐	For hierarchical and complex designs, identification of the appropriate level for tests and full reporting of outcomes
☒	☐	Estimates of effect sizes (e.g. Cohen's *d*, Pearson's *r*), indicating how they were calculated

Our web collection on statistics for biologists contains articles on many of the points above.

Software and code

Policy information about availability of computer code

Data collection	PatchMaster (HEKA)
Data analysis	pClamp 10 (Molecular Devices); Sigmaplot (SPSS); MATLAB (Mathworks); Pymol (Schrodinger);

For manuscripts utilizing custom algorithms or software that are central to the research but not yet described in published literature, software must be made available to editors/reviewers. We strongly encourage code deposition in a community repository (e.g. GitHub). See the Nature Research guidelines for submitting code & software for further information.

Data

Policy information about availability of data

All manuscripts must include a data availability statement. This statement should provide the following information, where applicable:
- Accession codes, unique identifiers, or web links for publicly available datasets
- A list of figures that have associated raw data
- A description of any restrictions on data availability

All relevant data collected, analyzed and shown in the figures, as well as input dataset and code for SCA analysis are available in source data.

Field-specific reporting

Please select the one below that is the best fit for your research. If you are not sure, read the appropriate sections before making your selection.

☒ Life sciences ☐ Behavioural & social sciences ☐ Ecological, evolutionary & environmental sciences

For a reference copy of the document with all sections, see nature.com/documents/nr-reporting-summary-flat.pdf

Life sciences study design

All studies must disclose on these points even when the disclosure is negative.

Sample size	Each electrophysiology experiment was performed on at least 3 individual cells. The number of recordings of each experiment was based on personal experience and data variability.
Data exclusions	Electrophysiology recordings displaying high noises, instability, or currents were contaminated by endogenous currents from oocytes, for example at very positive or negative voltages, were excluded.
Replication	All experiments were successfully reproduced.
Randomization	The experiments were not randomized.
Blinding	The investigators were not blinded to allocation during experiments and outcome assessment.

Reporting for specific materials, systems and methods

We require information from authors about some types of materials, experimental systems and methods used in many studies. Here, indicate whether each material, system or method listed is relevant to your study. If you are not sure if a list item applies to your research, read the appropriate section before selecting a response.

Materials & experimental systems

n/a	Involved in the study	
☐	☒	Antibodies
☒	☐	Eukaryotic cell lines
☒	☐	Palaeontology
☐	☒	Animals and other organisms
☒	☐	Human research participants
☒	☐	Clinical data

Methods

n/a	Involved in the study	
☒	☐	ChIP-seq
☒	☐	Flow cytometry
☒	☐	MRI-based neuroimaging

Antibodies

Antibodies used	For KCNQ1 (primary antibody conjugated with HRP, do not need secondary antibody) Name: KCNQ1 (G-8) mouse monoclonal IgG Cat. No.: sc-365186 Vendor: Santa Cruz.<br>For Gβ, Primary antibody, Name: Gβ (T-20) rabbit polyclonal IgG Cat. No.: sc-378 Vendor: Santa Cruz. Secondary antibody Name: Goat anti-Rabbit IgG (H+L) Secondary Antibody, HRP Cat. No.: A16110 Vendor: Thermo
Validation	Both KCNQ1 and Gβ bands are consistent with those provided from the vendor.

Animals and other organisms

Policy information about studies involving animals; ARRIVE guidelines recommended for reporting animal research

Laboratory animals	Xenopus laevis
Wild animals	n/a
Field-collected samples	Stage V or VI oocytes were obtained from Xenopus laevis by laparotomy. All procedures were approved by the Washington University Animal Studies Committee. In details, frogs werer housed in a professional animal facility in the basement of Whitaker Hall downstairs from the laboratory where experiments are performed. Frogs were cared for by the Institutional Animal Care and Use Committee Office at Washington University. Oocytes removal operations were performed in animal preparation rooms in the animal facility. Technical assistance will be provided by the animal facility.
Ethics oversight	All procedures were performed in accordance with the protocol approved by the Washington University Animal Studies Committee (Protocol # 20190030)

Note that full information on the approval of the study protocol must also be provided in the manuscript.
